# Validation of a rapid semi-automated method to assess left atrial longitudinal phasic strains on cine cardiovascular magnetic resonance imaging

**DOI:** 10.1186/s12968-018-0496-1

**Published:** 2018-11-05

**Authors:** Shuang Leng, Ru-San Tan, Xiaodan Zhao, John C. Allen, Angela S. Koh, Liang Zhong

**Affiliations:** 1 National Heart Research Institute Singapore, National Heart Centre Singapore, 5 Hospital Drive, Singapore, 169609 Singapore; 2 Duke-NUS Medical School, 8 College Road, Singapore, 169857 Singapore

**Keywords:** Cardiovascular magnetic resonance, Left atrial function, Strain, Strain rate

## Abstract

**Background:**

Abnormal left atrial (LA) function is a marker of cardiac dysfunction and adverse cardiovascular outcome, but is difficult to assess, and hence not, routinely quantified. We aimed to determine the feasibility and effectiveness of a fast method to measure long-axis LA strain and strain rate (SR) with standard cardiovascular magnetic resonance (CMR) compared to conventional feature tracking (FT) derived longitudinal strain.

**Methods:**

We studied 50 normal controls, 30 patients with hypertrophic cardiomyopathy, and 100 heart failure (HF) patients, including 40 with reduced ejection fraction (HFrEF), 30 mid-range ejection fraction (HFmrEF) and 30 preserved ejection fraction (HFpEF). LA longitudinal strain and SR parameters were derived by tracking the distance between the left atrioventricular junction and a user-defined point at the mid posterior LA wall on standard cine CMR two- and four-chamber views. LA performance was analyzed at three distinct cardiac phases: reservoir function (reservoir strain *ε*_*s*_ and strain rate SR_s_), conduit function (conduit strain *ε*_*e*_ and strain rate SR_e_) and booster pump function (booster strain *ε*_*a*_ and strain rate SR_a_).

**Results:**

There was good agreement between LA longitudinal strain and SR assessed using the fast and conventional FT-CMR approaches (*r* = 0.89 to 0.99, *p* < 0.001). The fast strain and SRs showed a better intra- and inter-observer reproducibility and a 55% reduction in evaluation time (85 ± 10 vs. 190 ± 12 s, *p* < 0.001) compared to FT-CMR. Fast LA measurements in normal controls were 35.3 ± 5.2% for *ε*_*s*_, 18.1 ± 4.3% for *ε*_*e*_, 17.2 ± 3.5% for *ε*_*a*_, and 1.8 ± 0.4, − 2.0 ± 0.5, − 2.3 ± 0.6 s^− 1^ for the respective phasic SRs. Significantly reduced LA strains and SRs were observed in all patient groups compared to normal controls. Patients with HFpEF and HFmrEF had significantly smaller *ε*_*s*_, SR_s_, *ε*_*e*_ and SR_e_ than hypertrophic cardiomyopathy, and HFmrEF had significantly impaired LA reservoir and booster function compared to HFpEF. The fast LA strains and SRs were similar to FT-CMR for discriminating patients from controls (area under the curve (AUC) = 0.79 to 0.96 vs. 0.76 to 0.93, *p* = NS).

**Conclusions:**

Novel quantitative LA strain and SR derived from conventional cine CMR images are fast assessable parameters for LA phasic function analysis.

**Electronic supplementary material:**

The online version of this article (10.1186/s12968-018-0496-1) contains supplementary material, which is available to authorized users.

## Background

Left atrial (LA) function has been increasingly recognized as an important determinant of cardiovascular morbidity and mortality [1]. The key role of LA is to modulate left ventricular (LV) filling through three phases [2]: 1) Reservoir phase – LA collects pulmonary venous return during LV contraction and isovolumetric relaxation; 2) Conduit phase – LA conducts blood passively into the LV; 3) Booster pump phase – atrial contraction that actively forces blood to the LV.

LA function can be assessed using LA pressure-volume loops [3], which require invasive measurements. Non-invasive assessment of dynamic changes in LA size, including diameters, areas and volumes, might be inadequate for describing complex LA phasic function [4]. Tissue Doppler imaging (TDI) has been used to measure late diastolic mitral annular velocity during atrial contraction for assessing LA function [5]. Errors arising from angle dependency mitigate its diagnostic accuracy and negate its utility [6]. Strain analyses have been performed using speckle-tracking echocardiography [7] or cine cardiovascular magnetic resonance (CMR) feature tracking (FT) [8]. However, the former is challenging for the LA due to low signal-to-noise ratio and the thin atrial wall; while the latter is hindered by the complex LA anatomy comprising the LA appendage and pulmonary veins [1]. Furthermore, lack of standardized methodology in strain analysis affects reproducibility of LA strain measurements [9]. Herein, we aimed to evaluate a novel and rapid semi-automatic post-processing method for assessing long-axis strain and strain rate (SR) for the determination of LA phasic longitudinal function from standard cine CMR images. The new method does not require total delineation of the LA contours, but only requires annotations of three distinct anatomical reference points, thus it is less affected by the presence of the LA appendage and pulmonary vein. We hypothesized that LA strain and SR derived with this novel method would require less processing time without compromising accuracy and reproducibility.

## Methods

### Study population

The study population consisted of 50 normal controls, and 130 patients (40 with heart failure (HF) with reduced ejection fraction (HFrEF, LV ejection fraction (EF) < 40%); 30 HF with mid-range ejection fraction (HFmrEF, LVEF 40–49%); 30 HF with preserved ejection fraction (HFpEF, LVEF ≥ 50%); and 30 patients with hypertrophic cardiomyopathy (HCM)). Inclusion criteria for HF required the presence of signs or symptoms of HF based on modified Framingham criteria [10] and prior hospitalization with primary diagnosis of HF. Exclusion criteria for HF included specific subgroups of HF (e.g., amyloidosis, eosinophilic myocarditis, etc.) and isolated right heart disease. HCM patients were recruited from specialized cardiomyopathy clinics. All patients had sinus rhythm during examination. The protocol was approved by the SingHealth Centralised Institutional Review Board and informed consent was obtained from all participants.

### CMR acquisition

CMR acquisitions were performed using a 3 T system (Ingenia, Philips Healthcare, Best, The Netherlands). Balanced steady steady state free precession (bSSFP) end-expiratory breath hold cine images were acquired in multi-planar long-axis views including the 2- and 4-chamber views. Typical parameters were as follows: TR/TE, 3/1 ms; matrix, 240 × 240; flip angle, 45°; field of view, 300 × 300 mm^2^; pixel bandwidth, 1776 Hz; pixel spacing, 1.25 × 1.25 mm; slice thickness, 8 mm; number of cardiac frames, 30/40 per cardiac cycle.

### Feature tracking (FT)

**Fig. 1:**
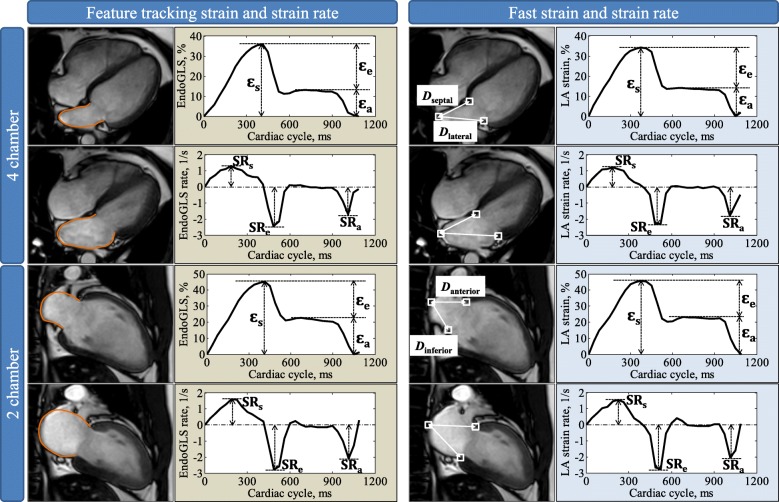
Left atrial (LA) strain and strain rate measurement in 4- and 2-chamber views. Left figures show an example of LA feature tracking and strain and strain rate profiles (Endo GLS: endocardial global longitudinal strain). Right figures show the fast long-axis strain and strain rates. White squares denote the three anatomical reference points that were tracked automatically throughout the cardiac cycle. *D* is the distance between the left atrioventricular junction and the user-defined point at the mid posterior LA wall on standard CMR 4-chamber (denoted as *D*_septal_ and *D*_lateral_) and 2-chamber (denoted as *D*_anterior_ and *D*_inferior_) views. Fast strain was derived from time variation of distance *D*. Strain rate was then obtained by taking the first-order derivative of the strain curve. The fast strain and strain rate curves on the right side are based on averaging the results from septal and lateral walls for 4-chamber view and from anterior and inferior walls for 2-chamber view, respectively. Reservoir strain (*ε*_*s*_) and strain rate (SR_s_) correspond to reservoir function. Conduit strain (*ε*_*e*_) and strain rate (SR_e_) correspond to conduit function. Booster strain (*ε*_*a*_) and strain rate (SR_a_) correspond to booster pump function. Details see text

With dedicated QStrain software (Version 2.0, Medis BV, Leiden, The Netherlands), FT was used to track tissue voxel motion on cine CMR images in deriving LA longitudinal strain and SR in both 2- and 4-chamber views. In each view, the LA endocardial contour was manually drawn when the atrium was at its minimum volume after atrial contraction. The length of the contour was then partitioned into a series of 48 evenly spaced points. Automatic contour tracking was then performed by tracking each single point based on a hierarchical algorithm that combined 1- (1D) and 2-dimensional (2D) tracking [11]. Manual contour adjustments were made at the frame when the LA volume was maximal. The LV end-diastolic and end-systolic phases coincide with the minimum and maximum LA volume phases with 30/40 cardiac frames per cycle that was employed in the cine CMR acquisition in this study. Tracking was repeated for three times and global LA longitudinal strain and SR measurements were averaged across all three repetitions in both 2- and 4-chamber views. Figure 1 (left column) shows a representative example of the LA tracking in the 2- and 4-chamber views and the strain data (i.e. the endocardial global longitudinal strain (GLS)). SR results were then obtained by taking the first-order derivative of the strain curve.

### Fast long-axis strain

Long-axis strain was assessed by automatically tracking the distance (*D*) between the left atrioventricular junction and a user-defined point at the mid posterior LA wall on standard CMR 2- and 4-chamber views (Fig. 1 right column). The atrioventricular junctions were selected as the mitral valve insertion points at the septal and lateral borders of the annulus on the 4-chamber view, and the anterior and inferior annular insertion points on the 2-chamber view [12–15]. The mid-point of posterior LA wall was defined as the intersection point of the LA posterior wall and the LA long-axis (the length of which is used for estimating the LA volume based on area-length formula) [16]. The tracking system in this study used the method of template matching [17]. Briefly, a small square (called mask) was manually drawn in the LV end-diastole frame containing the anatomical point of interest (white squares in Fig. 1 right column), and automatically tracked within a search region that was coincident with the mask and automatically selected by the program in the target frame, typically the next temporal frame in the cardiac cycle. A correlation map was produced using template matching algorithm based on normalized cross correlation, in which the point with highest correlation coefficient indicated the location of the best match. This point was used to update the mask in the target frame and the same procedure was automatically executed iteratively for all subsequent frames over the cardiac cycle. The point tracking was semi-automatic with mask selection in the initial frame as the only user input. Technical details including the processing parameters can be found in our prior study [14].

Longitudinal strain (*ε*) at any time point (*t*) in the cardiac cycle from LV end-diastole (time 0) was calculated as [18, 19]: *ε*(*t*) = (*D*(*t*) − *D*_0_) × 100/*D*_0_. Similar to previous definitions from FT-CMR [20, 21], LA reservoir strain (*ε*_*s*_), conduit strain (*ε*_*e*_) and booster strain (*ε*_*a*_) were calculated at *t* equal to LV end-systole, diastasis and pre-LA systole, respectively, and the corresponding peak SRs (SR_s_, SR_e_, SR_a_) were derived (Fig. 1). Strain and SR parameters were derived for each of the four measured walls (septal and lateral in 4-chamber view and anterior and inferior in 2-chamber view) and the average values were used for analysis.

### Left atrium volumetric analysis

LA volumetric analysis was performed using QMass software (Version 8.0, Medis BV). Apical 2- and 4-chamber views were reviewed offline to measure the maximal LA volume at LV end-systole (LAV_max_), diastasis LA volume at LV diastole before LA contraction (LAV_preA_), and minimal LA volume at LV end-diastole (LAV_min_) using biplane area-length method [22]: LA volume (ml) = 0.85*A_2C_* A_4C_/L, where A_2C_ and A_4C_ are the LA areas on the 2- and 4-chamber views, and L is the shorter long-axis length of LA in both 2- and 4-chamber views determined as the distance of the perpendicular line bisecting mitral annular plane that intersects with the posterior wall of the LA. LA phasic function, viz. reservoir, conduit and booster pump function were characterized by total LA emptying fraction (LAEF), passive LAEF and active LAEF, respectively, according to the following equations [20]:

$$ Total\ LAEF\ \left(\%\right)=\frac{\left({LAV}_{\mathrm{max}}-{LAV}_{\mathrm{min}}\right)\times 100}{LAV_{\mathrm{max}}} $$

$$ Passive\ LAEF\ \left(\%\right)=\frac{\left({LAV}_{\mathrm{max}}-{LAV}_{\mathrm{preA}}\right)\times 100}{LAV_{\mathrm{max}}} $$

$$ Active\ LAEF\ \left(\%\right)=\frac{\left({LAV}_{\mathrm{preA}}-{LAV}_{\mathrm{min}}\right)\times 100}{LAV_{\mathrm{preA}}} $$

### Statistical analysis

Statistical analysis was conducted using SPSS (version 17.0, International Business Machines, Armonk, New York, USA). Continuous data were summarized as mean ± SD. Comparisons of characteristics and strain data between patient groups and control subjects were performed using independent *t* tests for normally distributed data, Mann-Whitney *U* tests for non-normally distributed data, and chi-square tests for categorical data. Pearson’s *r* correlation, Passing-Bablok non-parametric regression, and Bland-Altman plots were used to assess the agreement of fast long-axis strain data and FT derived longitudinal strain. Correlation *r* larger than 0.7 was interpreted as a strong relationship between two variables. Pearson’s correlation coefficients were calculated to determine the linear association between LA strain and volumetric measurements. A *p* value less than 0.05 was considered statistically significant.

To evaluate the reproducibility, intra-observer and inter-observer variability were studied on a randomly selected subgroup of 20 cases (10 normal controls and 10 patients) using Bland-Altman analysis and coefficient of variation. For inter-observer variability, measurements were repeated by a second-independent observer, blinded to the first observer’s results. For intra-observer variability study, the analysis was repeated by the same observer who re-analyzed the same 20 cases after 1 week.

## Results

### Baseline characteristics of study population

Baseline demographics and clinical characteristics of controls (*n* = 50), all HF groups (HFrEF, *n* = 40; HFmrEF, *n* = 30; HFpEF, *n* = 30) and HCM patients (*n* = 30) are summarized in Table 1. All HF groups were highly symptomatic (69% New York Heart Association [NYHA] II-IV) with 83% of them on diuretics. There were no significant differences among the groups in age, gender and body surface area (BSA). Patients with HFrEF had significantly lower LVEF, larger LV end-diastolic volume and end-systolic volume indices, and smaller LAEF compared to the other groups (controls, HCM, HFpEF, HFmrEF). Patients with HCM, HFpEF and HFmrEF had significantly higher LA volumes and lower LAEF in all phases compared with normal controls. There were no significant differences between HCM and HFpEF with respect to LA volumes and LAEF.

**Table 1:** Baseline demographic and clinical characteristics of study subjects

Variables	Normal Controls (*n* = 50)	HCM (*n* = 30)	HFpEF (*n* = 30)	HFmrEF (*n* = 30)	HFrEF (*n* = 40)
Age, years	56 ± 13	55 ± 14	62 ± 11	57 ± 10	56 ± 10
Gender, Male/Female	34/16	15/15	23/7	20/10	29/11
BSA, m^2^	1.7 ± 0.2	1.8 ± 0.3	1.8 ± 0.2	1.9 ± 0.2	1.8 ± 0.3
DBP, mmHg	78 ± 10	76 ± 10	81 ± 24	76 ± 12	73 ± 14
SBP, mmHg	135 ± 18	137 ± 18	142 ± 26	134 ± 22	122 ± 19*#$
NYHA class	1.0 ± 0.0	1.2 ± 0.4	1.6 ± 0.5*#	1.8 ± 0.7*#	2.1 ± 0.9*#$
NT-pro-BNP, pg/mL^a^	–	–	229 (113, 608)	479 (304, 781)$	2079 (807, 4930)$^
Presence of MR (%)	0 (0%)	7 (23%)*	6 (20%)*	15 (50%)*	24 (60%)*#$
Medications (%)					
Diuretics	0 (0%)	6 (20%)*	21 (70%)*#	23 (77%)*#	39 (98%)*#
ACE inhibitor/angiotensin receptor blocker	0 (0%)	14 (47%)*	24 (80%)*#	22 (73%)*#	29 (73%)*#
β-Blocker	0 (0%)	24 (80%)*	23 (77%)*	20 (67%)*	36 (90%)*
Aspirin	0 (0%)	11 (37%)*	17 (57%)*	15 (50%)*	20 (50%)*#
LV EDV index, ml/m^2^	70 ± 11	76 ± 26	78 ± 16	87 ± 19*	138 ± 35*#$^
LV ESV index, ml/m^2^	26 ± 6	23 ± 16	34 ± 8*	50 ± 11*#$	105 ± 35*#$^
LV SV index, ml/m^2^	45 ± 8	53 ± 15*	44 ± 9#	38 ± 9#	33 ± 11*#$
LV EF, %	64 ± 6	72 ± 12*	56 ± 4*#	44 ± 3*#$	26 ± 8*#$^
LV Mass index, g/m^2^	48 ± 10	91 ± 23*	85 ± 39*	63 ± 19*#	82 ± 21*
LA volume index					
Max, ml/m^2^	36 ± 8	43 ± 11*	41 ± 12*	44 ± 20*	55 ± 16*#$^
Diastasis, ml/m^2^	26 ± 5	33 ± 9*	34 ± 11*	37 ± 18*	47 ± 15*#$^
Min, ml/m^2^	15 ± 4	22 ± 6*	22 ± 8*	28 ± 19*	40 ± 16*#$^
LA EF					
Total, %	59 ± 5	51 ± 7*	48 ± 7*	40 ± 13*#$	29 ± 13*#$^
Passive, %	27 ± 7	24 ± 7	19 ± 7*	19 ± 7*	14 ± 6*#$^
Active, %	43 ± 8	36 ± 7*	36 ± 8*	26 ± 11*#$	17 ± 11*#$^

Data are represented as mean ± SD (or ^a^median (interquartile range)). *BSA* body surface area, *DBP* diastolic blood pressure, *SBP* systolic blood pressure, *NYHA* New York Heart Association, *MR* mitral regurgitation, *LV* left ventricular, *EDV* end-diastolic volume, *ESV* end-systolic volume, *SV* stroke volume, *LVEF* left ventricular ejection fraction, *LA* left atrial, *LAEF* left atrial emptying fraction, *HCM* hypertrophic cardiomyopathy, *HFpEF* heart failure with preserved ejection fraction, *HFmrEF* heart failure with mid-range ejection fraction, *HFrEF* heart failure with reduced ejection fraction, *significant difference compared to controls, #significant difference compared to HCM, $significant difference compared to HFpEF, ^significant difference compared to HFmrEF. ^a^NT-pro-BNP results were not available for controls and HCM; NT-pro-BNP results were available in 20 HFpEF and 20 HFmrEF patients

### Fast LA strain measurements

**Fig. 2:**
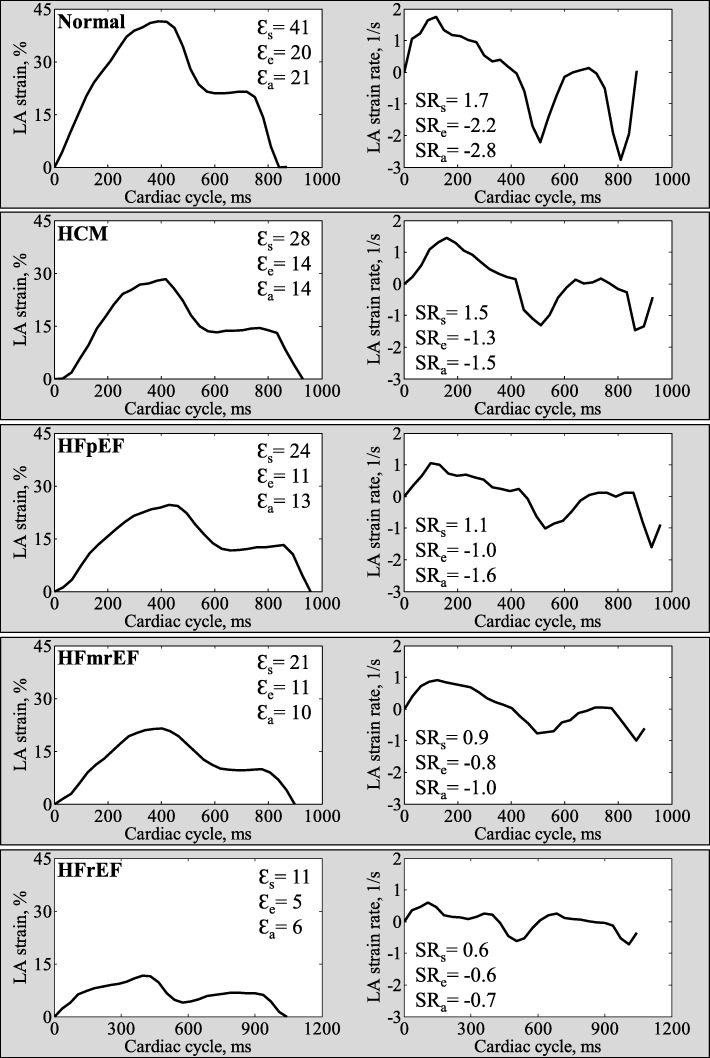
Representative examples of fast LA strain and strain rate in normal control, HCM, HFpEF, HFmrEF and HFrEF

Fast LA strain analysis was successfully performed in all subjects. Averaged strain and SR profiles were calculated from both the 2- and 4-chamber views. Figure 2 shows representative examples of fast LA strain and SR data in normal control, HCM, HFpEF, HFmrEF and HFrEF. Mean values in all normal controls were 35.3 ± 5.2% for reservoir strain, 18.1 ± 4.3% for conduit strain, 17.2 ± 3.5% for booster strain, and 1.8 ± 0.4, − 2.0 ± 0.5, − 2.3 ± 0.6 s^− 1^ for the respective phasic SRs. In comparison to controls, patients with HCM, HFpEF, HFmrEF and HFrEF had significantly decreased LA strain and SR measurements. Among the patients, HFrEF had lowest LA strain and SR compared to other patient groups (HCM, HFpEF, HFmrEF). Patients with HFpEF and HFmrEF had significantly smaller LA reservoir strain and SR, and conduit strain and SR than HCM, and HFmrEF had significantly impaired LA reservoir and booster function compared to HFpEF (Table 2). The strain and SR results derived by conventional FT-CMR are presented in Additional file 1: Table S1.

**Table 2:** Comparison of fast left atrial strain (*ε*) and strain rate (SR) measurements among subject groups

Parameters	Normal Controls (*n* = 50)	HCM (*n* = 30)	HFpEF (*n* = 30)	HFmrEF (*n* = 30)	HFrEF (*n* = 40)
Left atrial longitudinal strain					
Reservoir *ε*_*s*_, %	35.3 ± 5.2	27.0 ± 4.8*	23.8 ± 4.5*#	18.8 ± 6.6*#$	12.0 ± 5.8*#$^
Conduit *ε*_*e*_, %	18.1 ± 4.3	12.1 ± 4.3*	9.6 ± 3.2*#	8.6 ± 3.4*#	6.1 ± 3.1*#$^
Booster *ε*_*a*_, %	17.2 ± 3.5	14.9 ± 3.5*	14.2 ± 3.7*	10.2 ± 4.9*#$	6.0 ± 3.8*#$^
Left atrial longitudinal strain rate					
Reservoir SR_s_, 1/s	1.8 ± 0.4	1.3 ± 0.3*	1.1 ± 0.2*#	0.9 ± 0.3*#$	0.6 ± 0.2*#$^
Conduit SR_e_, 1/s	−2.0 ± 0.5	−1.1 ± 0.4*	−0.9 ± 0.3*#	−0.8 ± 0.3*#	−0.6 ± 0.3*#$^
Booster SR_a_, 1/s	−2.3 ± 0.6	−1.8 ± 0.4*	−1.7 ± 0.3*	−1.2 ± 0.5*#$	− 0.7 ± 0.4*#$^

Data are represented as mean ± SD. HCM: hypertrophic cardiomyopathy, *HFpEF* heart failure with preserved ejection fraction, *HFmrEF* heart failure with mid-range ejection fraction, *HFrEF* heart failure with reduced ejection fraction, *significant difference compared to controls, #significant difference compared to HCM, $significant difference compared to HFpEF, ^significant difference compared to HFmrEF

Strong correlations were exhibited between volumetric indices and fast LA strain/SR measurements for reservoir (*ε*_*s*_ vs. Total LAEF: *r* = 0.92; SR_s_ vs. Total LAEF: *r* = 0.83), conduit (*ε*_*e*_ vs. Passive LAEF: *r* = 0.83; SR_e_ vs. Passive LAEF: *r* = − 0.76) and booster pump functions (*ε*_*a*_ vs. Active LAEF: *r* = 0.88; SR_a_ vs. Active LAEF: *r* = − 0.84) (Table 3).

**Table 3:** Correlation of left atrial volumetric measurements and corresponding fast strain and strain rate parameters

Phases	Strain/SR	Volumetric measurements	Correlation coefficient	*P* value
Reservoir	*ε*_*s*_	Total LAEF	0.92	< 0.0001
	SR_s_	Total LAEF	0.83	< 0.0001
Conduit	*ε*_*e*_	Passive LAEF	0.83	< 0.0001
	SR_e_	Passive LAEF	−0.76	< 0.0001
Booster pump	*ε*_*a*_	Active LAEF	0.88	< 0.0001
	SR_a_	Active LAEF	−0.84	< 0.0001

*ε* left atrial strain, *SR* left atrial strain rate, *LAEF* left atrial emptying fraction

**Fig. 3:**
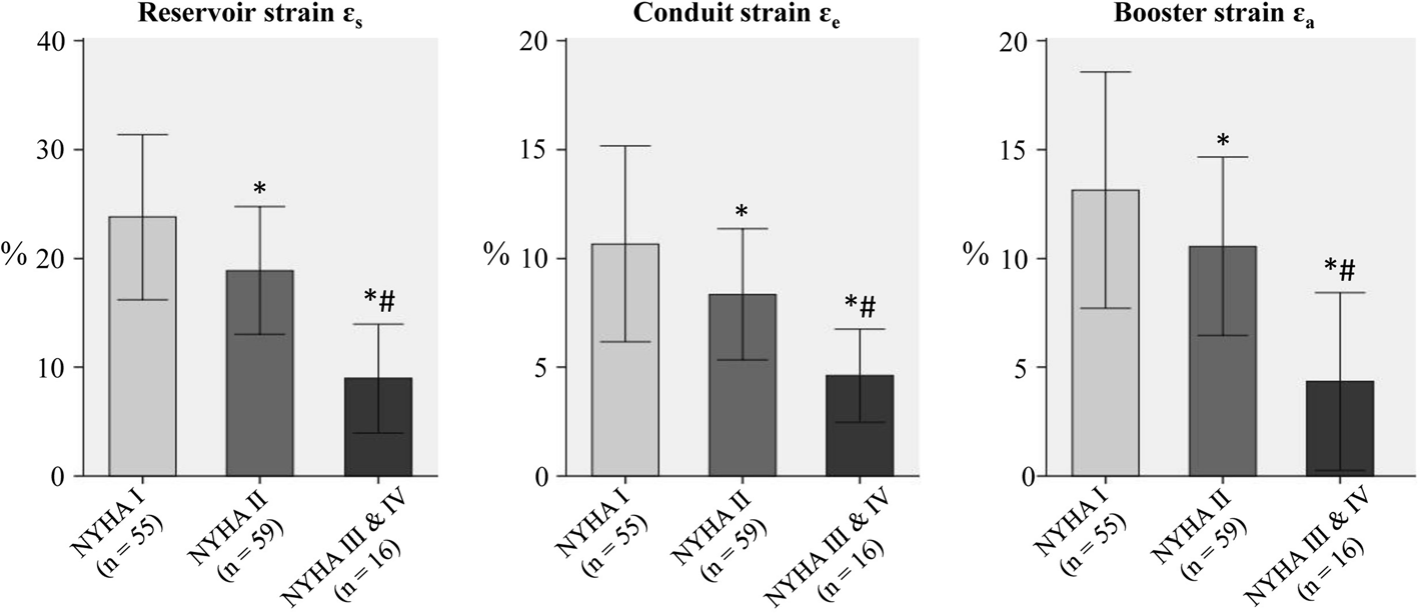
The effect of NYHA class on the fast LA strain in patient groups (HCM, HFpEF, HFmrEF, HFrEF). *p < 0.05 vs. NYHA I; #*p* < 0.05 vs. NYHA II

**Fig. 4:**
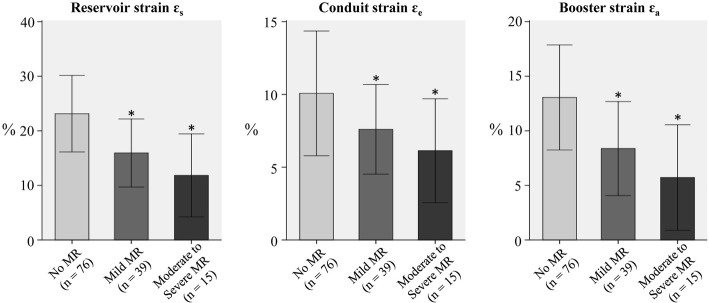
The effect of mitral regurgitation (MR) on the fast LA strain in patient groups (HCM, HFpEF, HFmrEF, HFrEF). *p < 0.05 vs. patients without MR

LA strain was negatively associated with NYHA class (Fig. 3) in the patient groups (HCM, HFpEF, HFmrEF, HFrEF). Significantly reduced fast LA strain measurements were also observed in patients with mitral regurgitation (*n* = 54) compared to patients without mitral regurgitation (*n* = 76) in the patient groups (HCM, HFpEF, HFmrEF, HFrEF) (Fig. 4).

**Fig. 5:**
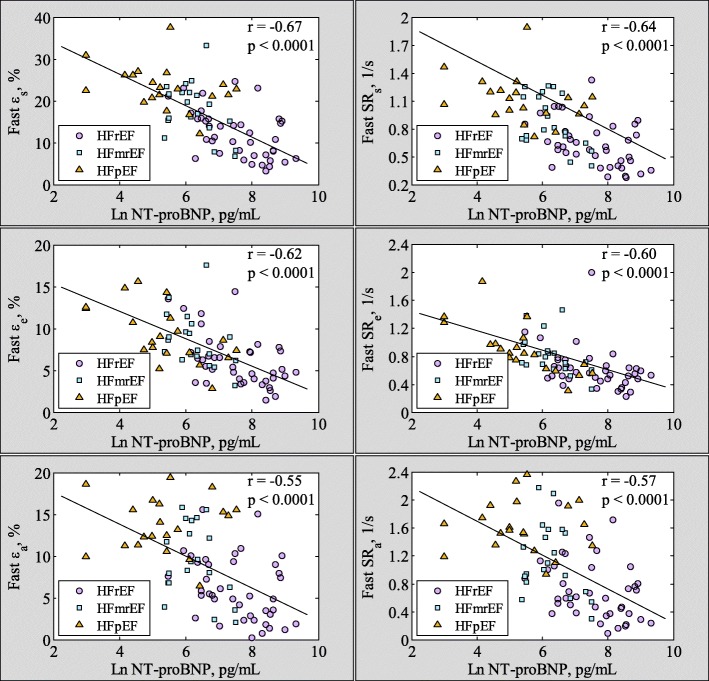
Correlation between fast LA phasic strain (left column)/strain rate (right column) and Ln NT-proBNP level in heart failure patients. Note here that absolute values of SR_e_ and SR_a_ were used in the correlation analysis. *NT-proBNP results were available in 20 HFpEF and 20 HFmrEF patients

Significant negative correlations were found between Ln NT-proBNP and fast *ε*_*s*_ (*r* = − 0.67, *p* < 0.001), *ε*_*e*_ (*r* = − 0.62, *p* < 0.001), *ε*_*a*_ (*r* = − 0.55, *p* < 0.001), SR_s_ (*r* = − 0.64, *p* < 0.001), SR_e_ (*r* = − 0.60, *p* < 0.001, absolute values were used for correlation analysis here) and SR_a_ (*r* = − 0.57, *p* < 0.001, absolute values were used for correlation analysis here) (Fig. 5) in HF patients.

### Comparison with feature tracking strain

**Fig. 6:**
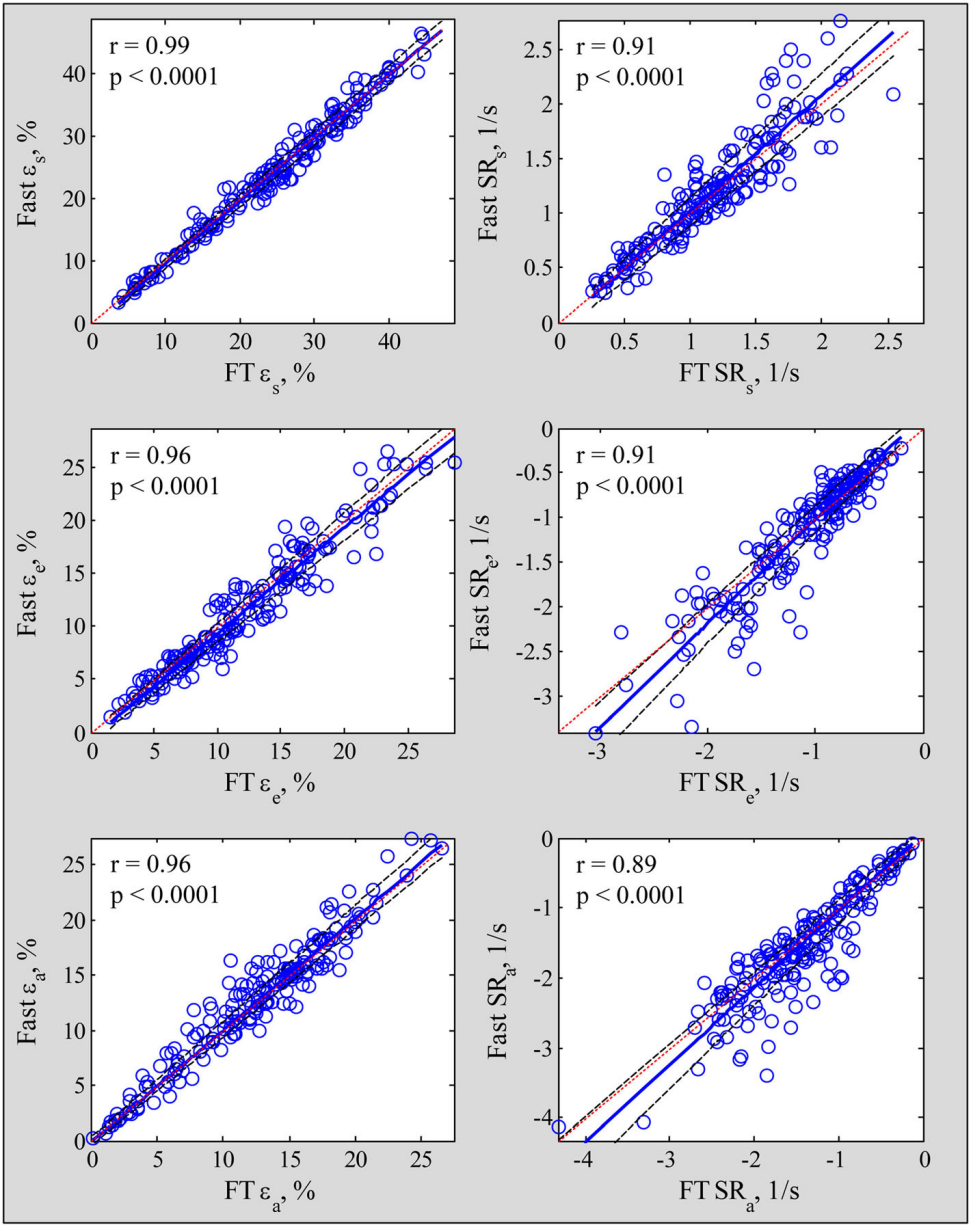
Correlation plots for the fast strain (left column) and strain rate (right column) compared with feature tracking (FT) derived measurements. Blue solid line and black dash lines denote Passing Bablok non parametric regression line and 95% confidence interval, respectively. Red dot line denotes equality line

**Fig. 7:**
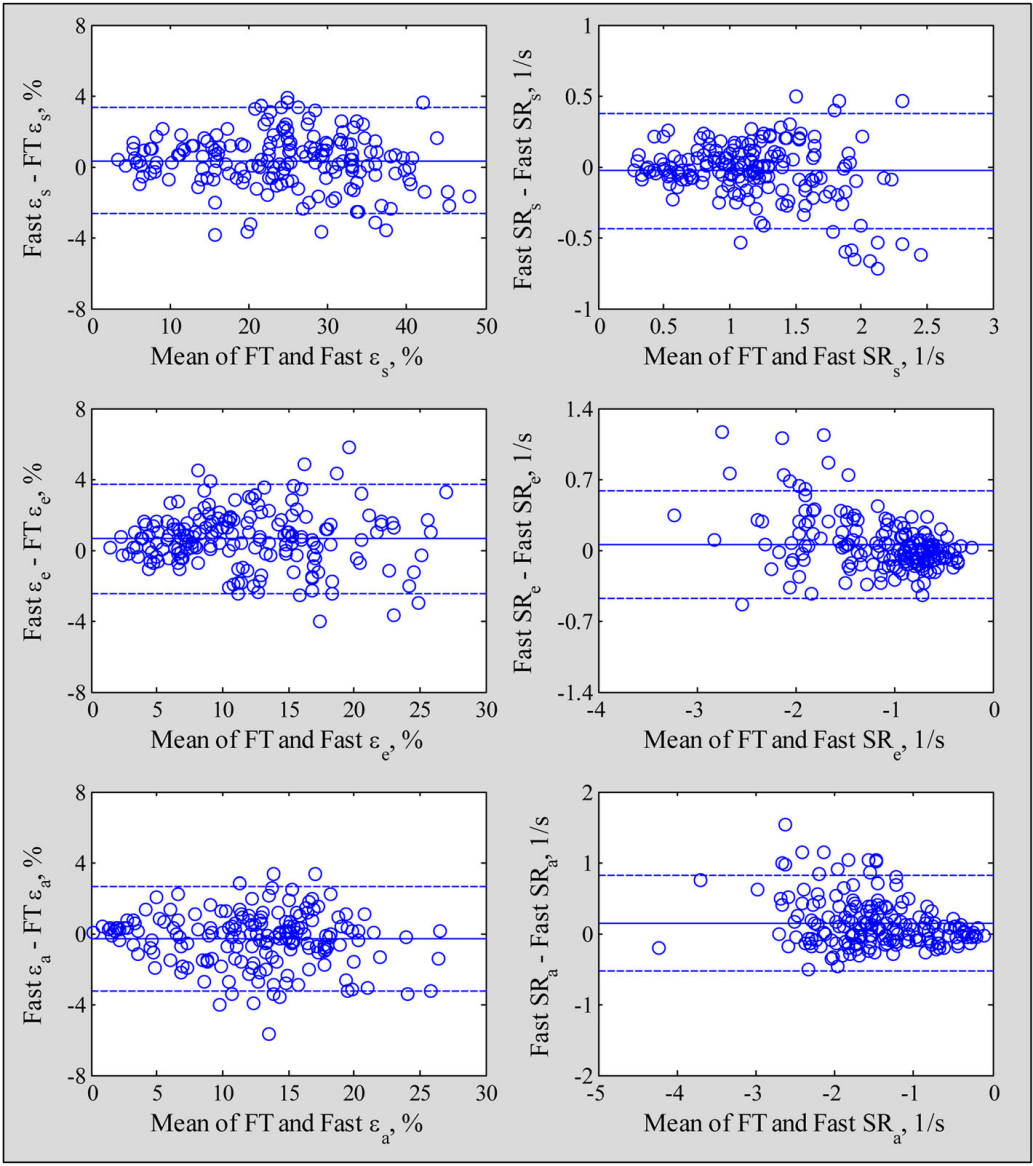
Bland-Altman plots for the fast LA strain (left column) and LA strain rate (right column) compared with feature tracking (FT) derived measurements. The bias (solid line) and limits of agreement (dash lines) are shown in each graph

Fast LA strain and SR measurements exhibited strong correlation and agreement with FT based strain derived from dedicated software (reservoir strain and SR, *r* = 0.99 and 0.91; conduit strain and SR, *r* = 0.96 and 0.91; booster strain and SR, *r* = 0.96 and 0.89) with low bias and narrow 95% limits of agreement (Figs. 6 and 7).

### Prediction of disease states

Table 4 reports results of the receiver operating characteristic (ROC) analysis performed, on a per subject basis, to assess the utility of LA volumetric and functional parameters for characterizing LA functional alterations in the patient groups (HCM, HFpEF, HFmrEF) compared with controls. Compared to LA volumetric and FT based strain parameters, fast LA strain and SR measurements characterizing LA alterations achieved higher levels of accuracy and greater areas under ROC curves (AUCs). The best variable for discriminating patients with HCM, HFpEF or HFmrEF from normal controls was fast LA SR for conduit phase (SR_e_) with AUC 0.96, sensitivity 93%, and specificity 90%.

**Table 4:** Utility of fast left atrial strain and strain rate parameters, as well as volumetric measurements to differentiate patients with HCM, HFpEF and HFmrEF from controls^a^

Variables	AUC	Sensitivity	Specificity	Threshold
Reservoir function				
Fast reservoir *ε*_*s*_, %	*0.94*	*0.79*	*0.98*	*27.6*
Fast reservoir SR_s_, 1/s	0.93	0.76	0.98	1.26
FT reservoir *ε*_*s*_, %	0.90	0.82	0.88	26.7
FT reservoir SR_s_, 1/s	0.84	0.80	0.80	1.24
Total LAEF, %	0.88	0.68	0.96	51.1
Conduit function				
Fast conduit *ε*_*e*_, %	0.92	0.76	0.96	12.4
Fast conduit SR_e_, 1/s	*0.96*	*0.93*	*0.90*	*−1.42*
FT conduit *ε*_*e*_, %	0.91	0.82	0.86	14.3
FT conduit SR_e_, 1/s	0.93	0.82	0.96	−1.14
Passive LAEF, %	0.76	0.75	0.74	24.3
Booster pump function				
Fast booster *ε*_*a*_, %	0.79	0.80	0.66	15.6
Fast booster SR_a_, 1/s	*0.85*	*0.76*	*0.82*	*−1.80*
FT booster *ε*_*a*_, %	0.76	0.65	0.84	14.3
FT booster SR_a_, 1/s	0.77	0.88	0.58	−1.94
Active LAEF, %	0.78	0.80	0.68	39.3

^a^Results of the receiver operating characteristic (ROC) analysis with area under the curve (AUC), sensitivity, specificity and threshold. *ε* left atrial strain, *SR* left atrial strain rate, *FT* feature tracking, *LAEF* left atrial emptying fraction. Italic values indicate the best predictors of phasic LA function as determined by AUC

### Reproducibility and time requirement

Table 5 shows the intra- and inter-observer variability. Intra-observer CV was 3.6–6.2% for fast LA strain and 6.4–7.9% for the SR measurements, with corresponding inter-observer CVs of 4.7–8.9% and 8.1–9.7%, respectively. The mean ± SD time per subject required for fast LA strain measurement in 2- and 4-chamber views was 85 ± 10 s, which was significantly shorter than the mean measurement time of 190 ± 12 s using the FT derived strain analysis which was also evaluated in the 2- and 4-chamber views.

**Table 5:** Intra- and inter-observer reproducibility for left atrial strain and strain rate measurements derived by fast and FT approach

Method	Strain/SR	Intra-observer (*n* = 20)		Inter-observer (*n* = 20)	
		Bias (limits of agreement)	CV, %	Bias (limits of agreement)	CV, %
Fast approach	*ε*_*s*_, %	−0.01 (−2.37, 2.34)	3.6	−0.15 (−3.16, 2.86)	4.7
	*ε*_*e*_, %	−0.15 (−1.81, 1.51)	5.3	0.10 (−1.58, 1.77)	5.3
	*ε*_*a*_, %	0.14 (−1.76, 2.04)	6.2	−0.25 (−2.93, 2.43)	8.9
	SR_s_, 1/s	0.03 (−0.18, 0.23)	6.5	0.06 (−0.24, 0.35)	9.7
	SR_e_, 1/s	−0.05 (− 0.29, 0.18)	7.9	− 0.03 (− 0.29, 0.23)	8.1
	SR_a_, 1/s	−0.01 (− 0.25, 0.24)	6.4	−0.03 (− 0.38, 0.32)	9.4
FT approach	*ε*_*s*_, %	0.16 (−5.39, 5.72)	8.6	0.21 (−5.29, 5.71)	8.5
	*ε*_*e*_, %	0.24 (−2.54, 3.02)	8.8	−0.17 (−3.18, 2.84)	9.5
	*ε*_*a*_, %	−0.08 (− 3.01, 2.87)	9.6	0.38 (− 3.26, 4.02)	11.9
	SR_s_, 1/s	0.03 (−0.28, 0.34)	9.5	0.07 (−0.25, 0.39)	10.0
	SR_e_, 1/s	−0.06 (− 0.36, 0.25)	10.3	0.08 (− 0.26, 0.42)	11.4
	SR_a_, 1/s	−0.03 (− 0.44, 0.37)	10.6	− 0.08 (− 0.62, 0.45)	13.9

*ε* left atrial strain, *SR* left atrial strain rate, *FT* feature tracking, *CV* coefficient of variation

## Discussion

The LA has previously been considered a neglected chamber. The interest in LA function evaluation has resurged over the recent years, with more focus on FT methods based on whole LA delineation that is, in our opinion, more suitable for ventricular function assessment. The LA wall is made up of circumferential and longitudinal muscular bundles. The former is arranged at the base of the atria, while the latter predominates at the parietal walls [23]. Barbier et al. revealed that LA reservoir function is primarily determined by longitudinal descent of the cardiac base and LA chamber stiffness [24]. In this study, we demonstrated the feasibility and effectiveness of a novel and rapid assessable parameter for the analysis of global longitudinal LA function using standard CMR cine images. Our results showed that the fast LA longitudinal strain correlated strongly with FT derived strain measurements, while sensitivity and specificity data were similar. Fast LA strain and SR performed better at differentiating diseased groups from normal controls than LA volumetric measurements with respect to LA reservoir, conduit and contractile booster pump function. Therefore, our approach could supplant conventional FT when the LA longitudinal function is being assessed.

The clinical significance of global longitudinal LA strain has been demonstrated in echocardiographic studies of patients with HF and HCM [5, 25, 26]. LA strain in HFpEF was associated with worsening NYHA functional class [25]. Echocardiographic speckle tracking-assessed LA longitudinal strain correlated with pulmonary capillary wedge pressure – and therefore LV filling pressure – in subjects with advanced systolic HF [26]. LA longitudinal strain and SR were shown to be sensitive discriminators of HCM, non-HCM LV hypertrophy and healthy controls [5]. Other than HF and HCM, impaired echocardiography-assessed LA longitudinal strain is also seen in other conditions – valvular heart diseases [27, 28], atrial stunning [29], and hypertension and diabetes [30]. Of note, LA longitudinal strain demonstrated prognostic utility in HF [31], aortic stenosis [32], and after myocardial infarct [33]. In a study of HFpEF patients, LA reservoir strain correlated negatively with adverse events, and was an independent predictor of the composite outcome of cardiovascular hospitalization or death [31].

### Fast LA strain vs. feature tracking strain

Instead of tracking 48 points on the contour line by the FT analysis, we presented a fast strain parameter that required the automatic tracking of only 3 anatomically discrete points, and demonstrated its feasibility and capability to characterize the phasic longitudinal LA function compared to the conventional FT-based strain and SR measurements. Our technique is analogous to [18], in which Riffel et al. assessed LV longitudinal function by using the long-axis strain derived from the distance of the epicardial apical border to the midpoint of the line connecting the mitral valve insertion points (LAS-epi/mid). The LAS-epi/mid showed a high correlation with conventional FT analysis results, and was non-inferior for discriminating patients with cardiomyopathies from healthy controls. Another similar approach [19] was applied to analyze long-axis strain in the LV by measuring the distances from the mitral valve insertions to the epicardial apex. They observed good correlation between long-axis strain – assessed with echocardiography and CMR – and infarct mass in patients with prior myocardial infarction. The authors in these two studies [18, 19] used manual tracking to measure the displacement of the mitral annulus between LV end-systolic and end-diastolic phases of the cardiac cycle, which yield only LV systolic strain parameters. Ours was the first study to apply semi-automatic tracking through all temporal phases of the cardiac cycle, which allowed for the dynamic evaluation of the complex phasic LA function.

FT in LA is more challenging and time-consuming than in the LV. The thin atrial wall, complex LA anatomy, presence of the LA appendage and pulmonary veins all influence tracking quality, and are potential sources of error [1]. In [20], the tracking quality was inadequate in 10.8% of total segments; while in another study [34], CMR images were not interpretable in 13% of the study population due to the failure of LA wall tracking. In our study, we observed that FT was not accurate in determining the valve annulus position in about 15% of cardiac frames, which vitiated the accuracy of FT-derived phasic LA strain and SR measurements. In contrast, fast LA strain analysis was successful in all study subjects with good reproducibility. The target feature was not correctly located automatically in less than 2% of total discrete points tracked. This was due to temporal blurring, and necessitated manual correction.

### LA function in patients with HCM, HFpEF, HFmrEF and HFrEF

This study examined LA structure and function in HF by volumetric and strain analyses in the three HF phenotypes. Compared with controls, all patients with HFpEF, HFmrEF and HFrEF displayed abnormal LA size and function characterized by increased LA maximal and minimal volume indices, decreased LAEF, and decreased phasic LA strains and SRs.

In general, LA function was less impaired in HFpEF and HFmrEF than in HFrEF. HFmrEF is a new category of HF, intermediate HFrEF and HFpEF [35]. Patients with HFmrEF had clinical characteristics that are more similar to those of HFpEF than HFrEF [36]. However, studies of LA structure and function in the HFmrEF cohort are lacking. We found progressively decreased fast LA strain and SR measurements in HFpEF, HFmrEF and HFrEF. In all HF groups, LA function was significantly associated with right heart function (i.e. right ventricular ejection fraction (RVEF), *r* = 0.73 for RVEF vs. reservoir strain *ε*_*s*_; *r* = 0.59 for RVEF vs. conduit strain *ε*_*e*_; and *r* = 0.64 for RVEF vs. booster strain *ε*_*a*_, Additional file 2: Figure S1); and negatively with NYHA class. These data revealed the important role of LA dysfunction in HF. LA function can be used to monitor HF progression, and plausibly, strategies to maintain or restore normal LA function can help to improve NYHA scores and mitigate progression of right heart dysfunction [37]. Significant negative associations between NT-proBNP levels and LA phasic functions were found in our study. On multivariate analysis, only LVEF (β = − 0.037, *p* = 0.001) and fast LA reservoir strain *ε*_*s*_ (β = − 0.073, *p* < 0.0001) persisted as significant predictors of NT-proBNP, indicating the incremental contribution of fast LA strain measurements in HF.

Decreases in reservoir and conduit function and increases in booster pump function in HCM patients have been reported previously [1, 38, 39]. In our study, fast LA strain analysis also revealed significantly decreased reservoir and conduit strains and SRs in HCM compared to controls. Booster strain and SR, however, were also decreased in our HCM patients, which differed from the findings in [20]. In a recent study, Kowallick et al. [40] characterized LA strain in HCM according to the extent of LV hypertrophy and fibrosis, and found that LA booster pump function was impaired in HCM with severe late gadolinium enhancement (LGE) only (LGE ≥ 20%). Indeed, our HCM subjects may represent those with more advanced disease. In our data, 67% (20/30) of the HCM patients had moderate to severe patchy fibrosis. In addition, 43% (13/30) of our HCM patients had significant left ventricular outflow tract (LVOT) obstruction, which has also been reported to negatively influence LA mechanics [41].

The current study found significant deteriorations in LA function with presence of mitral regurgitation. Among the subjects with NYHA class II-IV in the patient group (HCM and HF), patients with moderate and severe mitral regurgitation had LA reservoir strain *ε*_*s*_ reduced by 35% and 49%, respectively, compared to those with mild or no mitral regurgitation. The corresponding reductions were 25% and 40%, and 44% and 56% for LA conduit strain *ε*_*e*_ and booster strain *ε*_*a*_, respectively. Studies have shown that global peak LA longitudinal strain is inversely correlated with the degree of mitral regurgitation [27], which may possibly be explained by LA abnormalities such as myocyte hypertrophy, interstitial fibrosis and decreased metalloproteinase expression [42].

### Reproducibility

All fast strain and SR measurements demonstrated better intra- and inter-observer reproducibility than FT-CMR (Table 5). The tracking system in this study used the method of template matching, which is a semi-automatic algorithm for searching and finding the location of a template image within a larger image. The template selection in the initial frame is the only user input. Hence, the observed intra- and inter-observer variability are expected to be insignificant since operator dependence is relegated exclusively to variation in site selection of the atrioventricular junction points and the user-defined point at the mid posterior LA wall. Fast strain and SR were evaluated with the tracking of only three discrete spatially separated points, and were thus less affected by the presence of the LA appendage and pulmonary veins, which are the primary factors hindering effective endocardial tracking in conventional FT strain analysis.

Prior studies have investigated the impact of repeated measures on reproducibility of FT-CMR [43, 44] where the differences in intra- and inter-observer variability were assessed based on single and averaged measurements (two and three repetitions with subsequent averaging of results, respectively). It was found that averaging of the results of repeated analyses improves the reproducibility of LA strain measurements. Although the benefit of repeated measures is relatively low, considering that doubling or tripling of analysis times would be required [43, 45], FT-CMR analysis was performed three times in the current study in order to further maximize its reproducibility [20]. The evaluation time presented herein and the ~ 55% in time savings were based on single tracking (i.e. the average time of three repetitions). In addition, inter-vendor comparisons have been performed in prior studies [43, 44, 46] to determine differences in strain measurements between commercially available FT-CMR software packages (TomTec, Medis QStrain and Circle Cardiovascular Imaging). The inter-vendor agreement was reasonably good for LV global circumferential strain and longitudinal strain, but was lower in right ventricular global longitudinal and radial strains [44, 46]. The time for post-processing of a given case did not vary between the different types of software [43]. No prior studies could be found that assessed the inter-vendor agreement for LA strain measurements, therefore, further studies are warranted to determine the interchangeability of LA longitudinal strain and strain rate between different FT-CMR software solutions.

### Limitation and long-term prospects

The fast LA strain is an index of global long axis LA function in the longitudinal direction, and does not provide information on segmental and regional deformation, circumferential and radial strain, or myocardial strain outside the LA. In the assessment of LA phasic function, our proposed fast LA strain/SR measurement method using standard CMR cine images yields similar number and type of metrics as FT-based assessment using proprietary software [20], i.e. LA longitudinal reservoir, conduit and active strains and the corresponding SRs.

The reported experience of using FT-CMR LA strain parameter assessment for clinical diagnosis is quite recent, and encompasses cohorts of patients undergoing atrial fibrillation catheter ablation [8, 47, 48], implantable cardioverter-defibrillator implant [47, 49], community volunteers without cardiovascular disease at baseline [50, 51], patients with acute myocarditis [45], obesity [52], and Ebstein’s anomaly [53]. These studies established the feasibility and validity of LA strain parameter measurement using CMR; and in [51] found negative correlation between LA strain and LA fibrosis. Notably, none of the published studies investigated the role of FT-derived LA strain parameters for HF diagnosis.

We focused on the use of CMR for HF diagnosis and have demonstrated that the fast method for assessing phasic LA longitudinal strains and SRs was useful in discriminating subjects with HFrEF and HFpEF – including a recently described and guideline-adopted classification of HFmrEF [35] – as well as HCM, which shares morphological similarities with HFpEF (concentric LV hypertrophy). Our findings corroborated the results of prior echocardiographic studies [5, 25, 26], and underscore the clinical diagnostic utility of and rationale for measuring LA longitudinal strain using either echocardiography or CMR.

We believe our fast method is interchangeable with, and can eventually supplant FT for several reasons. First, in this study we performed both FT and fast method for LA strain and SR measurements among HF, HCM and normal subjects, and demonstrated high degrees of correlation (Fig. 6) and agreement (Fig. 7) between the two methods. Importantly, the fast method had higher intra- and inter-observer reproducibility with tighter coefficient of variation (Table 5). Second, the FT method based on whole chamber contour delineation is, in our opinion, more suited for LV function assessment rather than the LA, where LA appendage and pulmonary vein anatomy interrupt the LA wall outline and complicate contour tracing and frame-to-frame phasic FT. Third, the LA wall is made up of circumferential muscle fibres at the base of the atria and predominantly longitudinal muscular bundles at the parietal walls [23]. Whereas FT includes the basal segment at the posterior aspect of the LA in the chamber contour for calculation, our fast method excludes it – arguably more directly mimicking the function of the longitudinal muscle bundles, which along with LA chamber stiffness, determines LA reservoir function [24]. Fourth, our automatic algorithm dispenses with manual LA contour tracing, requires minimal operator input and significantly shorter processing time, which should garner wider access and acceptance than FT.

The long-term prospect is to make the analysis fully automated, by automatically detecting the three anatomical reference points based on deep learning based technique in a large dataset. Second, the present study focused primarily on assessment of LA longitudinal function. A similarly comprehensive approach for the right atrial (RA) long-axis strain represents a potentially researchable goal in the future for evaluation of RA function. Third, this approach can be applied to derive papillary muscle longitudinal strain by automatically tracking the distance between papillary muscle tips and the left atrioventricular junction points throughout the cardiac cycle. The papillary muscle tethering distance and strain have been demonstrated to have significant association with ischemic mitral regurgitation [54]. Fourth, prior study has revealed distinct associations between metabolic perturbations with LA phasic function in healthy aging population [55]. Future investigations using both imaging (e.g. our fast LA strain analysis) and molecular approach in the patient groups may help to identify mechanisms involved in cardiovascular diseases in specific cohorts. Further studies are warranted on the association between booster LA strain and LA ejection force [56], as the latter is a measure of atrial contractile function and plays an important role in LV diastolic filling.

## Conclusions

The presented fast LA strain and SR measurements represent reliable and expeditiously calculable parameters for quantifying longitudinal LA deformation using routine clinical cine CMR images without specific acquisition protocol or special software tools. Fast LA strain measurements exhibited high correlation with conventional LA FT strain analysis and were comparable in discriminating patients with HF and HCM from normal controls, were less affected by the LA appendage and pulmonary veins, and required significantly less processing time.

## Additional files

Additional file 1: **Table S1.** Comparison of feature tracking derived left atrial strain (*ε*) and strain rate (SR) measurements among subject groups. (PDF 193 kb)

Additional file 2: **Figure S1.** Linear relation between right ventricular ejection fraction (RVEF) and fast left atrial (A) reservoir strain, (B) conduit strain and (C) booster strain in patients with heart failure. (PDF 98 kb)

## Abbreviations

*ε*_*a*_: Booster strain
*ε*_*e*_: Conduit strain
*ε*_*s*_: Reservoir strain
2D: Two-dimensional
A2c: Apical two-chamber
A4c: Apical four-chamber
AUC: Area under ROC curve
BSA: Body surface area
bSSFP: Balanced steady state free precession
CMR: Cardiovascular magnetic resonance
EF: Ejection fraction
FT: Feature tracking
GLS: Global longitudinal strain
HCM: Hypertrophic cardiomyopathy
HF: Heart failure
HFmrEF: Heart failure with mid-range ejection fraction
HFpEF: Heart failure with preserved ejection fraction
HFrEF: Heart failure with reduced ejection fraction
L: Length
LA: Left atrium/left atrial
LAEF: Left atrial emptying fraction
LAV_max_: Maximal LA volume
LAV_min_: Minimal LA volume
LAV_preA_: LA volume before LA contraction
LGE: Late gadolinium enhancement
LV: Left ventricle/left ventricular
LVOT: Left ventricular outflow tract
NYHA: New York Heart Association
RA: Right atrium/right atrial
ROC: Receiver operating characteristic
SR: Strain rate
SR_a_: Booster strain rate
SR_e_: Conduit strain rate
SR_s_: Reservoir strain rate
TDI: Tissue Doppler imaging
