# Supplementary material for: Validation of a rapid semi-automated method to assess left atrial longitudinal phasic strains on cine cardiovascular magnetic resonance imaging
Source: J Cardiovasc Magn Reson. 2018 Nov 5;20:71. doi: 10.1186/s12968-018-0496-1 (PMC6219067; doi:10.1186/s12968-018-0496-1)
Supplement: Supplementary file 1 — Table S1. Comparison of feature tracking derived left atrial strain (ε) and strain rate (SR) measurements among subject groups. (PDF 193 kb) [file 12968_2018_496_MOESM1_ESM.pdf]

Additional file 1: Table S1. Comparison of feature tracking derived left atrial strain (ε) and strain rate (SR) measurements among subject groups.

**Parameters**	**Controls (n = 50)**	**HCM (n = 30)**	**HFpEF (n = 30)**	**HFmrEF (n = 30)**	**HFrEF (n = 40)**
**Left atrial longitudinal strain**					
Reservoir ε_s, %	34.9 ± 5.0	27.9 ± 5.1*	24.4 ± 4.9*	19.1 ± 6.9*#$	12.6 ± 5.9*#$^
Conduit ε_e, %	18.4 ± 4.3	12.6 ± 4.1*	10.9 ± 3.3*#	9.2 ± 3.6*#	6.7 ± 3.5*#$^
Booster ε_a, %	16.5 ± 3.2	15.3 ± 3.7	13.5 ± 3.7*	9.8 ± 4.8*#$	5.9 ± 3.6*#$^
**Left atrial longitudinal strain rate**					
Reservoir SR_s, 1/s	1.7 ± 0.3	1.3 ± 0.3*	1.1 ± 0.2*	0.9 ± 0.3*#	0.6 ± 0.3*#$^
Conduit SR_e, 1/s	-1.7 ± 0.5	-1.0 ± 0.4*	-0.9 ± 0.3*	-0.8 ± 0.2*	-0.7 ± 0.3*#$
Booster SR_a, 1/s	-2.0 ± 0.6	-1.7 ± 0.5*	-1.5 ± 0.5*	-1.2 ± 0.6*#	-0.7 ± 0.4*#$^

Data are represented as mean ± SD. HCM: hypertrophic cardiomyopathy; HFpEF: heart failure with preserved ejection fraction; HFmrEF: heart failure with mid-range ejection fraction; HFrEF: heart failure with reduced ejection fraction; *significant difference compared to controls; #significant difference compared to HCM; $significant difference compared to HFpEF; ^significant difference compared to HFmrEF.
