# Supplementary material for: Validation of a rapid semi-automated method to assess left atrial longitudinal phasic strains on cine cardiovascular magnetic resonance imaging
Source: J Cardiovasc Magn Reson. 2018 Nov 5;20:71. doi: 10.1186/s12968-018-0496-1 (PMC6219067; doi:10.1186/s12968-018-0496-1)
Supplement: Supplementary file 2 — Figure S1. Linear relation between right ventricular ejection fraction (RVEF) and fast left atrial (A) reservoir strain, (B) conduit strain and (C) booster strain in patients with heart failure. (PDF 98 kb) [file 12968_2018_496_MOESM2_ESM.pdf]

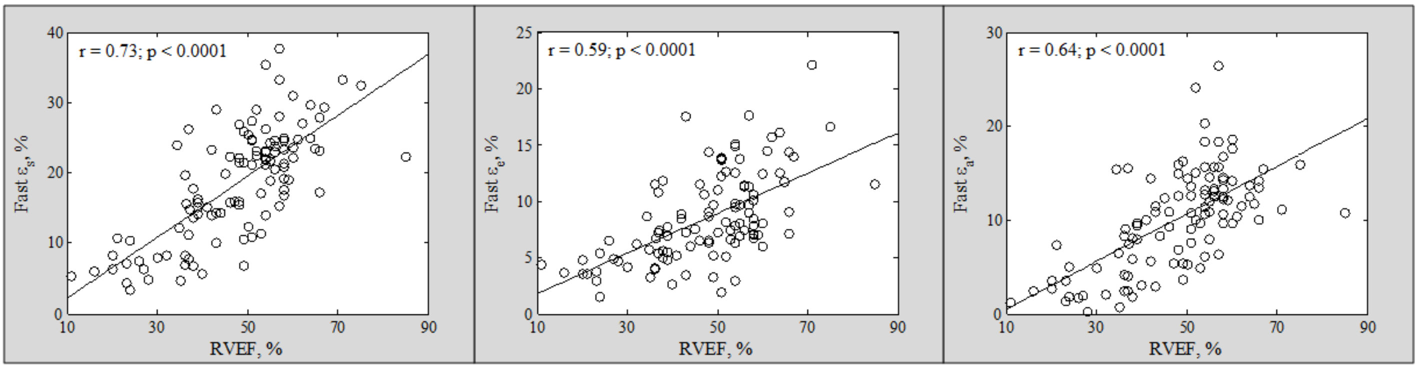

Additional file 2: Figure S1. Linear relation between right ventricular ejection fraction (RVEF) and fast left atrial (A) reservoir strain, (B) conduit strain and (C) booster strain in patients with heart failure.
